# Graft Remodeling following Transcrestal Sinus Floor Elevation via the Gel-Pressure Technique (GPT) and Pasteous Nano-Crystalline Hydroxyapatite Bone Substitute

**DOI:** 10.3390/ma8063210

**Published:** 2015-06-03

**Authors:** Bernhard Pommer, Ewald Unger, Dieter Busenlechner, Robert Haas, Georg Mailath-Pokorny, Rudolf Fürhauser, Georg Watzek

**Affiliations:** 1 Academy for Oral Implantology, Lazarettgasse 19/DG, Vienna 1090, Austria; E-Mails: busenlechner@implantatakademie.at (D.B.); haas@implantatakademie.at (R.H.); mailath@implantatakademie.at (G.M.-P.); fuerhauser@implantatakademie.at (R.F.); watzek@implantatakademie.at (G.W.); 2 Center for Medical Physics and Biomedical Engineering, Medical University of Vienna, Währinger Gürtel 18-20, AKH-4L, Vienna 1090, Austria; E-Mail: ewald.unger@meduniwien.ac.at

**Keywords:** dental implants, bone substitutes, sinus floor augmentation, maxillary sinus, bone transplantation, implant-supported dental prosthesis

## Abstract

Bone grafting of the maxillary sinus is attempted to compensate for sinus pneumatization and permit reliable insertion of endosseous dental implants for prosthetic rehabilitation. The aim of the present clinical investigation was to study bone regeneration four months after transcrestal sinus floor elevation via the Gel-Pressure Technique (GPT) and application of pasteous nano-crystalline hydroxyapatite bone substitute. A total of 25 patients with deficient alveolar ridges in the posterior maxilla (mean residual bone height: 4.7 ± 1.8 mm) were subjected to 32 flapless transcrestal sinus floor augmentations and simultaneous insertion of 40 implants. Sinus membrane elevation height averaged 11.2 ± 2.7 mm and minimal vertical graft resorption of 0.1 mm was observed after four months. Radiographic bone density averaged 460 Hounsfield units in regions adjacent to the native jawbone (1 to 7 mm distance), while reduction of bone density by −7.2%, −11.3%, −14.8%, −19.6% and −22.7% was recorded in more apical regions of 8, 9, 10, 11, and ≥12 mm distance to the original sinus floor, respectively. The results suggest that graft remodeling is completed up to a distance of 7 mm within a healing period of four months after sinus augmentation using nano-crystalline hydroxyapatite bone substitute material.

## 1. Introduction

Following postextraction alveolar bone resorption and pneumatization of the maxillary sinus cavity the quantity, as well as quality of available bone for dental implant placement in the edentulous posterior maxilla is frequently limited [1]. Internal bone augmentation of the maxillary sinus to compensate for sinus pneumatization and permit reliable insertion of endosseous implants for prosthodontic rehabilitation is based on the principle of guided bone regeneration using the sinus membrane as a natural barrier [2]. The formation of vital bone to allow for osseointegration of delayed or simultaneously placed implants is initiated by coronal displacement of the maxillary sinus mucosa (Schneiderian membrane) with or without addition of autologous bone and/or bone substitute material [3].

As described by Boyne in the 1960s, membrane elevation is accomplished via osteotomy of the lateral sinus wall, or else via a transcrestal approach to the antrum, as described by Summers in the 1990s [4]. Average survival rates reported in systematic reviews on dental implants placed in the grafted maxillary sinus range between 91.5% and 92.6% for the lateral approach, compared with mean survival rates between 93.5% and 96.4% following the transcrestal approach [5,6,7,8]. Comparisons are difficult to be made, however, due to relevant differences in confounding variables, such as residual bone quality and quantity, implant macrogeometry and surfaces, timing of implant placement and prosthetic loading, type of prosthesis and dentition of the opposing arch, patient-related determinants, as well as grafting materials or mixtures [9].

The transcrestal approach to the maxillary sinus is advocated as “minimally invasive” due to reduced postoperative morbidity and undisturbed vascularization of the graft material [10]. In addition to the conventional osteotome-mediated sinus floor elevation described by Summers in 1994 [11], various modifications of minimally invasive transcrestal sinus elevation surgery have been proposed: membrane elevation by inflation of a balloon catheter [12,13], the use of hydraulic pressure [14,15,16], negative pressure [17], or gel-pressure [18]. A great concern in transcrestal elevation techniques is the avoidance of iatrogenic sinus membrane perforation, as the elevation of the Schneiderian membrane is not performed under optical or tactile control [19]. Due to the limited access there is no possibility to repair the torn membrane without changing to a lateral surgical approach [20]. Perforation of the sinus membrane is particularly difficult to avoid in cases of maxillary sinus septa that can be found in 28.4% on sinuses, on average [21]. Another important question concerning minimally invasive sinus augmentation surgery is whether the obtainable amount of bone height is generally limited [22] and, therefore, a conventional lateral approach should be preferred in cases of severely resorbed maxillae [23]. The increase in bone height obtainable by transcrestal techniques has been shown to be inferior to the conventional lateral approach [24] and, as is true for most tactics to reduce surgical invasion, the planning phase prior to surgery becomes more important and time-consuming [25].

The Gel-Pressure Technique (GPT) was developed by George Watzek and his team in Vienna [18] and represents a minimally invasive procedure using flapless surgery. The keyhole approach inherently necessitates the application of pasteous bone substitute material through the narrow transcrestal osteotomy. The aim of the present clinical study was, thus, to investigate subsequent radiographic graft remodelling in relation to the distance to the original sinus floor following transcrestal sinus floor augmentation using a nano-crystalline hydroxyapatite bone substitute material.

## 2. Material and Methods

### 2.1. Patient Inclusion and Preoperative Workup

**Figure 1:**
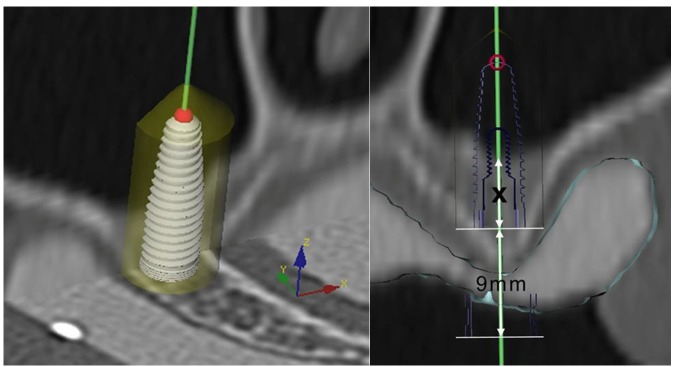
3D planning procedure for flapless transcrestal sinus floor elevation on preoperative CT scans to determine the correct drilling depth to avoid perforation of the maxillary sinus membrane.

Patients presenting with partial edentulism in the posterior maxilla (intermediate gaps or free end situations) and deficient bone height for dental implant placement (residual alveolar ridge lower than 7 mm) were included in the present prospective clinical study after obtaining written informed consent. The study protocol was approved by the responsible ethics committee of Vienna Medical University (EK 250/2007). CT scans were acquired with a conventional CT scanner (Tomoscan SR-6000, Philips, Eindhoven, The Netherlands) using a standard dental CT investigation protocol: 1.5 mm slice thickness, 1.0 mm table feed, 120 kV, 75 mA, 2 s scan time, 100–120 mm field of view, high-resolution bone filter [26]. Six radiopaque markers (gutta-percha balls) were placed into a polyvinyl siloxane impression of the edentulous jaw to perform the double-scan-technique [27,28]: The first scan was of the maxilla and the planning template *in situ*, the second of the planning template only. A computer assisted treatment-planning software (NobelClinician™, Nobel Biocare, Yorba Linda, CA, USA) allowed to superimpose the two sets of scans onto each other [29] and three-dimensionally plan the site of sinus trephination and implant position (Figure 1). The depth of the planned osteotomy was determined precisely in cross-sectional images at the elevation site to facilitate puncture of the bony sinus floor without perforation of the adherent sinus membrane. The planning data was then transferred to a dental laboratory and a custom surgical template with precision titanium tubes was fabricated.

### 2.2. Bone Graft and Implant Surgery

The surgical template was checked for proper seating and secured in place by 3 horizontal stabilization pins. A soft tissue punch of 4.1 mm diameter was carried out at the planned elevation site without mucoperiostal flap retraction. Cannon drills of 3.3 mm diameter (Friadent, Mannheim, Germany) with internal irrigation [30] were used for transcrestal osteotomies to puncture the bony floor of the sinus. Drill depth stops were applied to reduce the length of the cannon drill to the preplanned drilling depth, however, if no bony opening was created in the sinus floor by the first osteotomy the drilling depth was increased by 0.5 mm until puncture of the bony sinus floor was accomplished. The integrity of the sinus mucosa was then evaluated by direct visual examination, as well as the Valsalva maneuver.

**Figure 2:**
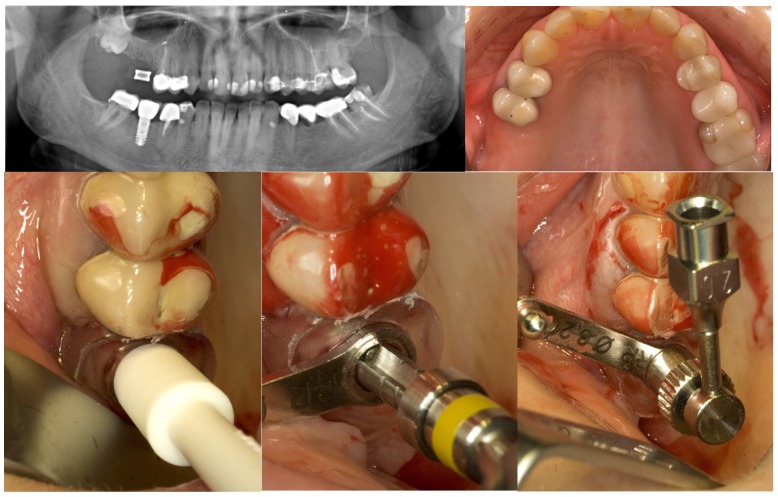

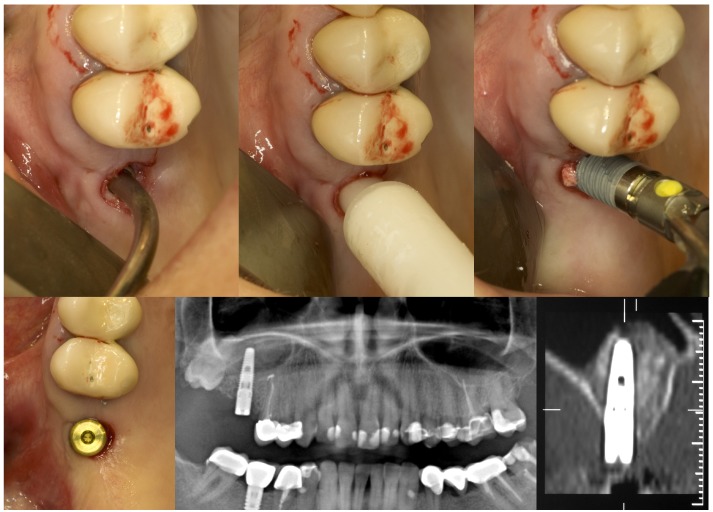
GPT and implant placement in the right upper first molar region of a 43-year old woman.

Thereafter a specially designed injection nozzle was inserted into the osteotomy and positioned 1 mm underneath the bony sinus floor. A silicon seal ring at the tip of the nozzle was compressed by rotation of a screw nut to tightly obturate the osteotomy and secure the nozzle in place. Under controlled pressure a radiopaque gel was administered through the injection nozzle to separate and elevate the Schneiderian membrane from the bony sinus floor until a total postoperative alveolar height of at least 13 mm was attained. Pressure control was achieved by a standard syringe to inject the gel gently with a low speed. After the integrity of the maxillary sinus membrane was evaluated on an intraoperative periapical x-ray, the radiopaque gel was washed out. Equal amounts of nano-crystalline hydroxyapatite bone substitute material (Ostim^®^, Heraeus Kulzer GmbH, Hanau, Germany) were administered through the transcrestal osteotomy and implants were placed and subjected to transmucosal healing (Figure 2).

### 2.3. Evaluation and Statistical Analysis

Alveolar bone height was assessed on preoperative and postoperative CT scans. Radiographic bone density was computed in Hounsfield Units (HU) in a distance of 1 mm apical to the original sinus floor up to the top of the sinus graft using the Easy Vision Workstation (Philips, Eindhoven, The Netherlands). Association of bone density to patient age and gender was tested using Spearman correlation coefficient and Wilcoxon signed rank test with continuity correction. Friedman rank sum including post hoc Nemenyi testing was performed to determine differences in bone density with regards to the distance to the original sinus floor at a significance level of p = 0.05. To determine a critical threshold, multiple Wilcoxon signed rank tests were performed at a Bonferroni-corrected significance level of p = 0.05/16 = 0.0032 to account for multiple testing. All analyses were performed using R-project software (R Foundation for Statistical Computing, Vienna, Austria).

## 3. Results

A total of 25 patients (12 women and 13 men) with a mean age of 45.6 ± 12.0 years and deficient alveolar ridges in the posterior maxilla (mean residual bone height: 4.7 ± 1.8 mm, range: 2.0–6.8 mm) were included. Flapless transcrestal sinus floor augmentation was performed in 32 sinuses via the Gel-Pressure Technique (GPT) and pasteous nano-crystalline hydroxyapatite bone substitute material [18]. In a bimodal approach, a total of 40 implants (Replace Select™, Nobel Biocare, Gothenburg, Sweden) with a mean length of 11.0 ± 1.4 mm (range: 10–13 mm) and a mean diameter of 4.3 ± 0.4 mm (range: 3.5–5.0 mm) were placed and subjected to transmucosal healing. No provisional loading was applied and impressions for final restorations were taken after at least four months of healing.

Integration of bone grafts and implants within the first four months after surgery was satisfactory in all patient cases. Intra-operative complications included one case of sinus membrane perforation resulting in abortion of the procedure (1/33 = 3.0%). Postoperative morbidity involved transient maxillary sinusitis in four patients (12.5%) that was successfully treated with oral antibiotics. Mean bone graft height was 11.2 ± 2.7 mm resulting in a mean postoperative bone height of 16.3 ± 2.8 mm. After four months of bone healing the mean bone height measured 16.2 ± 2.8 mm (mean vertical resorption of 0.1 mm) with only three cases of 1 mm graft shrinkage (9.4%) and two cases of 0.5 mm graft shrinkage (6.3%).

**Figure 3:**
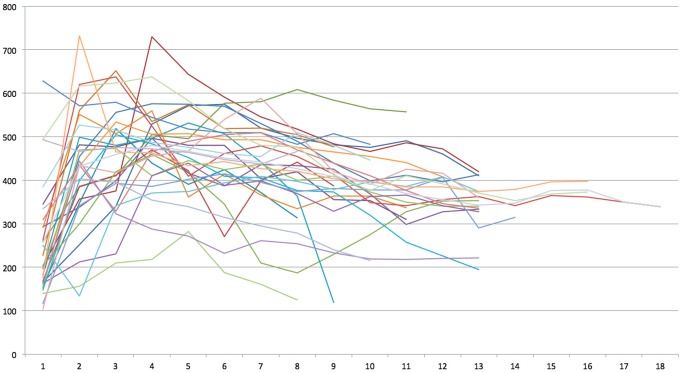
Bone density measurements of 32 transcrestal sinus bone grafts after 4 months of healing yielded between 104 and 755 Houndsfield Units (HU) in 1–18 mm distance to the sinus floor.

Mean radiograhic bone density per sinus measured 410.2 ± 78.2 HU (range: 186–541 HU) and did not differ between female (403.9 ± 82.9 HU) and male (415.7 ± 73.3 HU) participants (p = 0.681). No correlation between overall bone density and patient age could be observed (r_s_ = −0.05, p = 0.770). Radiographic bone density measured 256.8 ± 121.8 HU in 1 mm distance to the sinus floor, 431.8 ± 130.6 mm in 2 mm distance, 459.5 ± 110.3 HU in 3 mm distance, 480.2 ± 98.1 HU in 4 mm distance, 465.6 ± 92.4 HU in 5 mm distance, 450.7 ± 111.4 HU in 6 mm distance, 442.2 ± 113 HU in 7 mm distance, 426.3 ± 120.9 HU in 8 mm distance, 407.8 ± 108.7 HU in 9 mm distance, 391.8 ± 80.3 HU in 10 mm distance, 369.5 ± 85.6 HU in 11 mm distance, 355.9 ± 80.6 HU in 12 mm distance, 342.3 ± 60.5 HU in 13 mm distance, 347.3 ± 23.0 HU in 14 mm distance, 376.3 ± 14.0 HU in 15 mm distance, 377.0 ± 14.9 HU in 16 mm distance, 350 HU in 17 mm distance, and 339 HU in 18 mm distance (Figure 3).

A statistically significant difference in bone density depending on the distance to the original sinus floor was observed overall (Friedman χ^2^ = 98.9, df = 12, p < 0.001), however, *post hoc* Nemenyi testing revealed decrease in bone density between 8 mm and 11 mm (p < 0.001), exclusively, while no differences were observed between 1 and 7 mm and between 12 and 18 mm, respectively. Wilcoxon test regarding various thresholds yielded p-values of 0.1671 for distance ≤5 mm *vs*. >5 mm, p = 0.0132 for ≤6 mm *vs*. >6 mm, p = 0.0010 for ≤7 mm *vs*. >7 mm, and p = 0.0004 for ≤8 mm *vs*. >8 mm. Considering the Bonferroni-corrected significance level of p = 0.0032, a significant decrease of bone density was thus observed in a distance of more than 7 mm to the native jawbone.

## 4. Discussion

The results of the present prospective clinical study suggest that graft remodeling following transcrestal sinus floor elevation and augmentation using pasteous bone substitute material of nano-crystalline hydroxyapatite composition may be completed up to a distance of 7 mm to the original bony sinus floor within a healing period of four months. Significantly reduced radiographic bone density measurements were obtained in further apical areas of the sinus graft: 7.2% reduction in a distance of 8 mm to the residual alveolar ridge, 11.3% reduction in a distance of 9 mm, 14.8% reduction in a distance of 10 mm, 19.6% reduction in a distance of 11 mm, and 22.7% reduction in a distance of 12 to 18 mm to the original sinus floor (Table 1).

**Table 1:** Mean bone density in Houndfield Units (HU) in 1 to 18 mm distance to the residual alveolar ridge and percentage of reduction in bone density.

Distance to the Sinus Floor	Mean Bone Density	Percentage of Reduction
1 to 7 mm	459.6 HU	
8 mm	426.3 HU	–7.2%
9 mm	407.8 HU	–11.3%
10 mm	391.8 HU	–14.8%
11 mm	369.5 HU	–19.6%
12 to 18 mm	355.4 HU	–22.7%

These findings suggest that graft remodelling is completed up to a distance of 7 mm into the sinus graft within four months, while the area between 8 and 11 mm shows reduced graft remodelling with continuous decrease of radiographic bone density from −7.2% in 8 mm distance to −19.6% in 11 mm distance. By contrast, no differences could be observed in the most apical area of the bone graft between 12 and 18 mm from the sinus floor showing a mean reduction in bone density of −22.7%. This finding may be interpreted as reduced ossification in this area of the bone graft within four months of healing following bone augmentation surgery.

Graft shrinkage, *i.e.*, reduction of vertical bone height, was only 0.1 mm within the first four months after bone grafting. This compared to mean reduction in the height of transcrestal sinus grafts of 0.3 mm after three months and no less than 1.2 mm after 12 months in other investigations [31], however, graft shrinkage may heavily depend on the type of autogous bone or substitute material applied [32]. The mean gain in bone height, *i.e.*, the height of the graft material, was 11.2 mm in the present investigation and, thus, higher than reported with conventional transcrestal elevation techniques using osteotomes that has been reported to range between 2.5 and 8.6 mm [10,16,19,20,33,34]. A recent study, however, could show that membrane perforation rates are higher in thicker (≥3 mm), as well as thinner sinus membrane (≤0.5 mm) and, therefore, the achievable gain in bone height may be related to the condition of the Schneiderian membrane [35] along with other patient- and sinus-related factors, such as the presence of maxillary sinus septa, decreased residual bone height, and smoking [36].

The data presented are in line with the results of a preclinical study in 10 minipigs using the same pasteous nano-crystalline hydroxyapatite bone substitute [37] that investigated the “bridging distance” in 0 to 1 mm distance to the host bone, the “region of graft consolidation” in 2 to 3 mm distance, and the “region of ceasing bone formation” in 4 to 5 mm distance: after 12 weeks, the bone volume was 53.3%, 26.4%, and 5.8% and the bone substitute volume was 6.2%, 24.5%, and 41.2%, respectively, in the three defined regions. While comparison seems limited due to difference in study design, the percentage decrease in histologic bone volume (−47.5%) appears greater than the percentage decrease of radiographic bone density in the present investigation (−22.7%) due to the fact that pasteous bone substitute material presents with a certain amount of inherent radiographic density, even though no bone formation has occurred yet. The major limitation of the present study is, thus, the lack of information regarding the amount of newly formed bone. This may only be possible via bone biopsy that is hard to justify ethically in one-stage minimally invasive transcrestal sinus floor augmentation surgery.

## 5. Conclusions

Evaluation of radiographic bone density following transcrestal sinus floor elevation using pasteous nano-crystalline hydroxyapatite bone substitute suggests that graft remodeling is completed up to a distance of 7 mm to the original bony sinus floor within a healing period of four months. These results from transcrestal sinus floor augmentation may, however, not necessarily be applicable to lateral access sinus grafts and the space created may not necessarily represent bone formation. To generate sufficient bone volume in two-stage surgery (delayed implant placement) a residual bone height of 6 mm would thus be required for an implant length of 13 mm, respectively, a residual bone height of 3 mm for an implant length of 10 mm. In cases of even lower residual alveolar ridges a healing period longer than four months may prove advantageous, however, this needs to be investigated in clinical trials of two-stage bone grafting that allows for bone biopsy at the time of delayed implant placement. Short dental implants may be an alternative in cases of residual alveolar bone height of at least 5 mm.
